# Associations of adolescent social media use trajectories with spatial and verbal memory: a prospective cohort study

**DOI:** 10.1016/j.lana.2026.101454

**Published:** 2026-03-20

**Authors:** Jason M. Nagata, Jennifer H. Wong, Kristen E. Kim, Sahana Nayak, Elizabeth J. Li, Racquel A. Richardson, Andreas M. Rauschecker, Leo Sugrue, Kyle T. Ganson, Timothy Piatkowski, Jinbo He, Alexander Testa

**Affiliations:** a Division of Adolescent and Young Adult Medicine, Department of Pediatrics, University of California, San Francisco, 550 16th Street, Box 0503, San Francisco, CA, 94143, USA; b Department of Radiology & Biomedical Imaging, University of California, San Francisco, 505 Parnassus Ave, San Francisco, CA, 94143, USA; c Factor-Inwentash Faculty of Social Work, University of Toronto, 246 Bloor St W, Toronto, ON, M5S 1V4, Canada; d School of Applied Psychology and Griffith Centre for Mental Health, Griffith University, Brisbane, Australia; e Centre for Health Services Research, The University of Queensland, Brisbane, Australia; f Department of Biosciences and Bioinformatics, School of Science, Xi'an Jiaotong-Liverpool University, 215123, Suzhou, Jiangsu, China; g Department of Management, Policy and Community Health, University of Texas Health Science Center at Houston, 7000 Fannin St, Houston, TX, 77030, USA

**Keywords:** Social media, Screen time, Digital media, Adolescent health, Youth, Cognition

## Abstract

**Background:**

Evidence on screen time and cognition is mixed, with few longitudinal studies on social media patterns and memory. This study aimed to examine how social media trajectories relate to cognitive performance in early adolescence.

**Methods:**

We analyzed a prospective cohort (N = 7528, 51.1% male, mean age: 10 years (8–13 years), 41.8% non-White) from the Adolescent Brain Cognitive Development (ABCD) Study (baseline (2016–2018) to Year 2 (2018–2020)). Group-based trajectory modeling estimated patterns of daily social media use from baseline–Year 2. Three social media time trajectories: (1) no or very low use, (2) low but increasing use, and (3) high and increasing use were identified. Cognitive functioning was measured using the Little Man Task (LMT) and the Rey Auditory Verbal Learning Test (RAVLT). Linear regression models estimated the association between social media time trajectories and cognitive functioning at Year 2, adjusting for baseline age, sex, race, ethnicity, household income, parent education, attention-deficit/hyperactivity symptoms, depressive symptoms, respective baseline cognitive measures, non-social media screen use, and study site.

**Findings:**

Compared to no or very low social media use, low increasing social media use was associated with lower performance on the RAVLT Initial Trials (B: −1.38; 95% CI: −1.82, −0.94), RAVLT Retroactive Interference Trial (B: −0.38; 95% CI: −0.52, −0.25), and RAVLT Long Delay Trial (B: −0.41; 95% CI: −0.55, −0.26). Compared to no or very low social media use, high increasing social media use was associated with lower accuracy on the LMT (B: −0.03; 95% CI: −0.05, −0.01), and lower performance on the RAVLT Initial Trials (B: −1.90; 95% CI: −2.76, −1.04), RAVLT Retroactive Interference Trial (B: −0.61; 95% CI: −0.89, −0.32), and RAVLT Long Delay Trial (B: −0.55; 95% CI: −0.84, −0.27).

**Interpretation:**

Increases in social media time were prospectively associated with lower cognitive performance two years later. Monitoring digital use, implementing a Family Media Use Plan, and balancing screen time with cognitively enriching activities may help mitigate these effects. Future studies should examine the effects of various contemporary media on cognitive functioning.

**Funding:**

The research was supported by the Doris Duke Foundation (2022056).

**Research in context**

**Evidence before this study**

We searched PubMed and Google Scholar for studies on social media use and cognitive outcomes in adolescents, published in any language up to June 16, 2025, using terms including “social media,” “screen time,” “digital media,” “adolescent,” “youth,” “cognition,” “memory,” “learning,” “attention,” “executive function,” “verbal memory,” and “spatial memory.” This search was supplemented by reviewing references in identified articles and our own knowledge of the literature. In the revision stage, another literature search was conducted in November 2025 to capture recent publications. Our review identified several cross-sectional and longitudinal studies examining associations between social media engagement and cognitive outcomes. However, most studies relied on single-timepoint assessments or small, demographically limited samples, and few investigated specific longitudinal patterns of social media use in relation to performance on tasks measuring spatial processing or verbal memory, such as the Little Man Task or Rey Auditory Verbal Learning Test. Furthermore, many studies did not account for sociodemographic factors, baseline cognitive performance, or non-social media screen use, limiting the ability to characterize temporal associations between social media trajectories and cognitive development. Consequently, large-scale, diverse, prospective analyses of social media use patterns and their associations with adolescent cognitive outcomes remain scarce.

**Added value of this study**

This study provides critical knowledge regarding the longitudinal associations between social media use and cognitive development in early adolescence. Unlike prior research that relied on single-time point assessments and smaller sample sizes, this study utilizes trajectory-based modeling with a diverse national sample of 7582 US adolescents to capture patterns of use over time, highlighting how sustained or increasing social media engagement may influence broader cognitive functioning, including memory, learning, and visuospatial processing. Prior research has focused on general screen time, while this study offers new insight specifically regarding social media time. These study findings are more generalizable and robust compared to previous cross-sectional research due to the methodological approach and larger sample size. The prospective findings linking higher social media use and lower cognitive scores may shed light on the directionality of the association between social media and adolescent brain development.

**Implications of all the available evidence**

Patterns of social media use during early adolescence may affect overall cognitive functioning, including memory, attention, and learning. Monitoring digital behaviours and encouraging balanced activities that support mental engagement could help protect cognitive development. Paediatricians can support families to implement a ‘Family Media Use Plan’ to mitigate the risks associated with social media use. Future studies should seek to collect larger samples of high social media users to evaluate potential sociodemographic differences between use groups and examine how social media content affects the brain's biological structure and function by utilizing neurocognitive outcomes via neuroimaging.

## Introduction

Social media has become an increasingly prevalent part of the lives of early adolescents, as approximately 40% of 8- to 12-year-old children report social media usage.^1^ In 2023, the U.S. Surgeon General released an advisory highlighting the limited research on the impact of social media on youth mental health.^2^ The link between increased social media use and depressive symptoms in early adolescents^3^ suggests the possibility of other associations, such as a correlation between social media use and cognitive function. Given previously established associations between depressive symptoms and cognitive impairment, some researchers have suggested that depression may mediate the association between social media use and cognitive function; however, current evidence is limited and largely inconclusive.^4^ Prior studies have proposed that screen time and social media use may alter neurodevelopment in socially relevant regions, though these are preliminary findings and require further investigation.^5^^,^^6^ Research suggests that active parental mediation can help decrease excessive social media use and lower overall time spent online.^7^

Screen time use is categorized as television viewing, video game use, computer/internet use, and social media use.^8^ Overall, general screen time use has been more widely studied, while social media use remains relatively under-researched despite its growing prevalence. Though screen media usage in children can have both positive and negative effects on development, excessive exposure has been linked to poorer executive functioning, impaired sensorimotor development, and diminished cognitive performance in later years.^8^ A study of preschool-aged children (19–60 months) revealed a negative association between total screen time and working memory.^9^ Additionally, a systematic review of existing reviews observing a broad range of screen time effects on health and well-being found a weak association between social media and cognition, primarily due to the sparse body of prior literature, which can offer evidence-based insights regarding the associations between the two variables.^10^ Although prior studies have explored social media use, few have examined how usage patterns over time predict cognitive performance across multiple domains, highlighting the need for dedicated research that places cognition as a central outcome.

Prior studies have predominantly highlighted the effects of social media or screen time on brain development through the utilization of magnetic resonance imaging to examine structural changes related to brain growth and development.^5^^,^^6^ In contrast, our study seeks to examine possible associations between social media and cognitive function by analysing the development of learning processes such as visuospatial attention and verbal encoding. Existing literature on this topic yields mixed results, with studies typically focusing on older age groups or non-representative samples.^11^ Furthermore, many studies focus on overall screen time rather than distinguishing the specific effects of social media use.^12^^,^^13^ The present study also utilizes a unique social media trajectory modeling pattern, which enables the characterization of social media use patterns over time. This highlights how different groups may be associated with different outcomes; specifically, the ways in which social media use differentially develops over a three-year period during early adolescence, and whether those who use it more heavily over time have worse cognitive outcomes than those who do not. Trajectory modeling remains underutilized in similar studies, underscoring another gap in the literature.

In order to address these gaps, our study aims to examine the prospective associations between social media time and cognitive measures two years later, amongst a socio-demographically diverse, large sample of early adolescents. We hypothesize that greater time spent on social media will be associated with lower cognitive performance in participants.

## Methods

### Study population

In this cohort study, we analyzed data from baseline assessments (2016–2018, 8–11 years old) to 2-year follow-up assessments (2018–2020, 10–13 years old) of the Adolescent Brain Cognitive Development (ABCD) Study, an ongoing longitudinal study of adolescent health and cognitive development in 11,962 children recruited from 21 sites across the United States at baseline.^14^ By Year 1, 11,219 participants were retained, and by Year 2, 10,973 participants were retained.^15^ Attrition may be higher in the current analysis due to participants missing data, as outlined below. Recruitment and sampling of participants primarily occurred through educational institutions, with careful consideration given to factors such as sex, race and ethnicity, and socioeconomic status to attenuate potential biases in sample selection. Further details about the ABCD Study's measures, participants, and recruitment have been previously published.^16^

The study utilized data from the ABCD 5.1 release. Participants with missing Youth Screen Time Survey from baseline to Year 2 (n = 2333), cognition assessments at baseline and Year 2 (n = 1372), baseline mental health (n = 1), and baseline sociodemographic (n = 728) data were excluded, resulting in a final sample of 7528 adolescents (Figure S1). Participants who were missing one or more of these key measures were not included in the analysis. The differences in sociodemographic characteristics between included and excluded participants are outlined in Table S1. The sample size was calculated to ensure sufficient power to detect small-to-medium effects, accounting for anticipated attrition.^16^

### Ethical approval

Centralized institutional review board approval was granted by the University of California, San Diego (160091), and individual recruitment sites. Written informed consent was obtained from parents/guardians, while written assent was obtained from participants.

### Independent variable: youth-reported social media time

We assessed screen time data collected by the ABCD Youth Screen Time Survey at baseline (2016–2018), Year 1 (2017–2019), and Year 2 (2018–2020). Participants reported the hours per day spent using social media (e.g., Instagram, Facebook, Twitter) separately for weekdays and weekends.^17^ We performed a weighted average calculation of the participants’ typical weekday and weekend social media consumption to obtain a typical weekly measure ([weekday average x 5] + [weekend average x 2])/7, utilizing a previously used measure.^18^ The weighted average enabled us to quantify average daily social media use over a one-week period as a continuous outcome (hours/day). Supporting the validity of self-reported screen time, prior work has found it to be significantly positively correlated (r = 0.49; P < 0.001) with objectively measured screen use captured by a passive smartphone sensing app.^19^

### Dependent variables: Little Man Task and Rey Auditory Verbal Learning Test

Cognitive performance variables were assessed at Year 2 (2018–2020). The Little Man Task (LMT) measures visuospatial attention, perspective-taking, and mental rotation across 32 trials, following one practice trial. Although originally developed for use in adult samples,^20^ preliminary analyses from the ABCD Study suggest it may be valuable for detecting age-related changes in adolescents.^21^ During the task, participants observe a male figure holding a briefcase either in the right or left hand, with the figure positioned in one of four positions: upright, upside down, facing the participant, or facing away. Participants respond by pressing a button to indicate which hand is holding the briefcase. Accuracy is calculated as the proportion of correct responses, with values ranging between 0 and 1.

The Rey Auditory Verbal Learning Test (RAVLT) evaluates verbal encoding, learning, and memory recall.^21^ Respondents hear a list of 15 unrelated words (List A) and attempt to recall them across five learning trials. Consistent with methods used in prior ABCD analyses of RAVLT measures, the total number of words correctly recalled across the five initial learning trials was summed to produce an auditory verbal learning score, ranging from 0 to 75.^22^ After the five learning trials, a distractor list (List B) of 15 words is introduced and recalled, followed by a short delay recall of List A to evaluate retroactive interference. To evaluate longer-term retention, List A is recalled again after a 30-minute delay involving unrelated non-verbal tasks. The total number of words correctly recalled in the short delay recall and long delay recall is separately summed to produce memory recall scores, ranging from 0 to 15.

### Covariates

Several baseline covariates were included based on a directed acyclic graph (DAG) to account for sociodemographic, contextual, and behavioural factors that may plausibly confound associations between social media time and cognitive performance. These included age; sex (female, male); race and ethnicity (ascertained by parent or guardian report and categorized as Asian, Black, Hispanic or Latino, Native American, White, or other); annual household income (<$25,000, $25,000–$49,999, $50,000–$74,999, $75,000–$99,999, $100,000–$199,999, and ≥$200,000); highest parent education level (high school or less vs college or more); and study site. These factors have been consistently associated with both digital media use patterns and cognitive development in prior research.^23^, ^24^, ^25^, ^26^, ^27^

We also adjusted for participants’ baseline performance on each corresponding cognitive test to account for pre-existing differences in cognition and reduce the potential for regression to the mean. Attention-deficit/hyperactivity symptoms and depressive symptoms were included as covariates because both have been prospectively associated with social media use in the ABCD Study and are independently related to cognitive performance.^3^^,^^12^^,^^28^, ^29^, ^30^, ^31^ Symptoms were assessed using parent-reported Child Behavior Checklist (CBCL) measures reflecting behaviour over the past six months, with T-scores calculated according to standard scoring procedures.^32^

Finally, non-social media screen time was included as a covariate to account for broader digital media exposure that may correlate with both social media use and cognitive outcomes.^33^, ^34^, ^35^ Non-social media screen time was calculated by summing average weighted daily use of watching television or movies, online videos (e.g., YouTube), playing video games, texting, and video chatting (e.g., FaceTime or Skype), and was winsorized at 16 h per day to limit the influence of extreme values.

### Statistical analysis

Social media time trajectories were created using group-based trajectory modeling (GBTM). GBTM is a finite mixture model that classifies individuals into a set number of latent groups based on the trajectory of a repeated measure.^36^ In the current study, GBTM was used to identify distinct latent patterns of average daily social media time among adolescents across three time points: baseline, Year 1, and Year 2. GBTM models were estimated using the “traj” package in Stata version 18.0.^37^ We tested two model structures, each estimating 2 to 4 trajectories: (1) trajectories modelled as a function of age without covariates, and (2) trajectories modelled as a function of age while adjusting for sex. The optimal model structure and number of trajectory groups were assessed using the Bayesian Information Criterion (BIC).^38^ Subsequently, the functional form of each trajectory (e.g., zero-order, linear, quadratic, or cubic) was refined for an optimal fit. The best-fitting model was selected based on the BIC and sufficient sample size in each trajectory. The optimal model identified was a sex-adjusted three-group solution comprising one linear trajectory and two cubic trajectories (Table S2).

After classifying participants into a given trajectory group, multiple linear regression analyses were used to estimate the association between baseline–Year 2 social media time trajectory and Year 2 cognitive performance scores, adjusting for covariates. In addition, sex-stratified cognitive performance outcomes were examined (Table S4). All analyses were conducted with sampling weights to approximate the American Community Survey by the US Census. The testing was 2-sided, and P < 0.05 was considered statistically significant. The Benjamini-Hochberg procedure was used to adjust for a false discovery rate given multiple statistical tests.^39^

### Role of the funding source

The funding sources had no role in the study design; in the collection, analysis, and interpretation of data; in the writing of the report; or in the decision to submit the paper for publication.

## Results

In our sample (N = 7528), the average age was 10 years old (standard deviation [SD] = 0.6), 48.9% (3617/7528) were female, and 51.1% (3911/7528) were male (Table 1). Our sample was racially and ethnically diverse, with 58.2% (4350/7528) identifying as White, 5.3% (455/7528) Asian, 14.2% (1240/7528) Black, 18.1% (1159/7528) Latino/Hispanic, 3.1% (267/7528) Native American, and 1.1% (57/7528) identifying as other races. Compared to the included participants, excluded participants were more likely to be from racial or ethnic minority groups, households with lower incomes, and to have parents with lower levels of education (Table S1). Participants demonstrated statistically significant sociodemographic differences across social media trajectory groups for sex, race and ethnicity, household income, and parent education (Table S3). The mean (SD) accuracy for the Little Man Task was 0.6 (0.2) at baseline and 0.7 (0.2) at Year 2. The baseline mean (SD) total correct scores were 44.8 (9.6) for the RAVLT Initial Learning Trials, 9.8 (3.0) for the RAVLT Retroactive Interference Trial, and 9.4 (3.1) for the RAVLT Long Delay Trial. The Year 2 mean (SD) total correct scores were 44.2 (9.1) for the RAVLT Initial Learning Trials, 9.7 (2.7) for the RAVLT Retroactive Interference Trial, and 9.1 (2.9) for the RAVLT Long Delay Trial. Compared to males, females had marginally higher mean cognitive performance scores across all measures at baseline and Year 2 (Table S4).

**Table 1:** Characteristics of the included participants in the Adolescent Brain Cognitive Development (ABCD) Study (N = 7528).

Characteristics	Mean (SD)/No. (%)
Age (years) (baseline)	10.0 (0.6)
Sex	
Female	3617 (48.9%)
Male	3911 (51.1%)
Race and ethnicity	
Asian	455 (5.3%)
Black	1240 (14.2%)
Latino/Hispanic	1159 (18.1%)
Native American	267 (3.1%)
Other	57 (1.1%)
White	4350 (58.2%)
Household income (baseline)	
$24,999 or less	886 (14.9%)
$25,000–$49,999	1019 (19.3%)
$50,000–$74,999	1053 (18.3%)
$75,000–$99,999	1175 (14.5%)
$100,000–$199,999	2479 (24.9%)
$200,000 or greater	916 (8.1%)
Parent's highest education (baseline)	
High school education or less	907 (14.7%)
College education or more	6621 (85.3%)
Child Behavior Checklist (baseline)	
Attention-deficit/hyperactivity symptoms (t-score)	53.2 (5.6)
Depressive symptoms (t-score)	53.7 (5.8)
Social media use trajectory (baseline–Year 2)	
No/very low	4289 (54.7%)
Low increasing	2822 (38.8%)
High increasing	417 (6.4%)
Non-social media screen time (hours/day) (baseline)	3.8 (3.1)
Cognitive performance measures (baseline)	
Little Man Task: Accuracy	0.6 (0.2)
Rey Auditory Verbal Learning Test: Initial Learning Trials	44.8 (9.6)
Rey Auditory Verbal Learning Test: Retroactive Interference	9.8 (3.0)
Rey Auditory Verbal Learning Test: Long Delay Recall	9.4 (3.1)
Cognitive performance measures (Year 2)	
Little Man Task: Accuracy	0.7 (0.2)
Rey Auditory Verbal Learning Test: Initial Learning Trials	44.2 (9.1)
Rey Auditory Verbal Learning Test: Retroactive Interference	9.7 (2.7)
Rey Auditory Verbal Learning Test: Long Delay Recall	9.1 (2.9)

Sampling weights based on the American Community Survey were used to represent population estimates. Participant counts are presented unweighted.

**Fig. 1:**
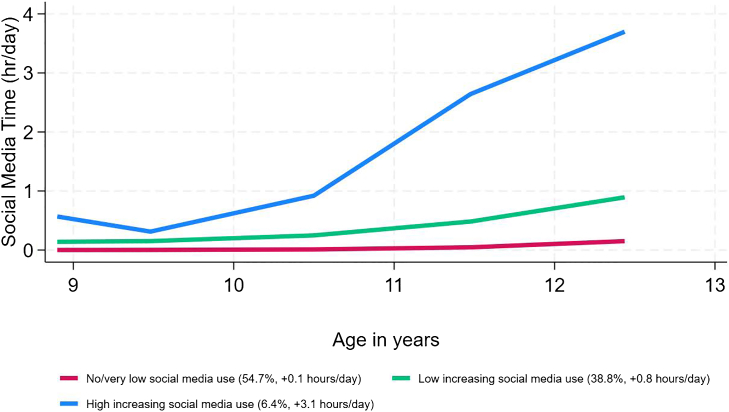
Social media time trajectories by age (Legend: Trajectory group (proportion of sample, change in daily social media time (hours/day) from baseline to Year 2)).

Results found three trajectory groups: no or very low social media use, low but increasing social media use, and high and increasing social media use (Fig. 1). The no or very low social media use group comprised 54.7% (4289/7528) of respondents. This group was characterized by an average of 0.1 h per day of social media use by age 13. Next, the low but increasing social media use group included 38.8% (2822/7528) of respondents. This group used an average of 0.8 h of social media per day by age 13. Finally, the smallest group was the high but increasing group, which comprised 6.4% (417/7528) of respondents. Participants in this group used an average of 3.1 h of social media per day by age 13. The following findings are comparisons with the reference group, no or very low social media use. Low but increasing social media use was associated with lower performance on the RAVLT Initial Trials (B [Beta]: −1.38; 95% CI [Confidence Interval]: −1.82, −0.94; P < 0.001), RAVLT Retroactive Interference Trial (B: −0.38; 95% CI: −0.52, −0.25; P < 0.001), and RAVLT Long Delay Trial (B: −0.41; 95% CI: −0.55, −0.26; P < 0.001) (Table 2). High and increasing social media use was associated with lower accuracy on the LMT (B: −0.03; 95% CI: −0.05, −0.01; P = 0.004), and lower performance on the RAVLT Initial Trials (B: −1.90; 95% CI: −2.76, −1.04; P < 0.001), RAVLT Retroactive Interference Trial (B: −0.61; 95% CI: −0.89, −0.32; P < 0.001), and RAVLT Long Delay Trial (B: −0.55; 95% CI: −0.84, −0.27; P < 0.001). The coefficients represent adjusted differences in Year 2 cognitive performance across trajectory groups rather than change over time.

**Table 2:** Associations between social media and cognition in the Adolescent Brain Cognitive Development (ABCD) Study (N = 7528).

Social media time trajectory	Little Man Task: Accuracy		Rey Auditory Verbal Learning Test: Initial Learning Trials		Rey Auditory Verbal Learning Test: Retroactive Interference		Rey Auditory Verbal Learning Test: Long Delay (30-min)	
	B (95% CI)	P	B (95% CI)	P	B (95% CI)	P	B (95% CI)	P
No/very low use (n = 4289)	Reference		Reference		Reference		Reference	
Low increasing (n = 2822)	−0.01 (−0.01, 0.003)	0.19	**−1.38 (−1.82, −0.94)**	**<0.001**	**−0.38 (−0.52, −0.25)**	**<0.001**	**−0.41 (−0.55, −0.26)**	**<0.001**
High increasing (n = 417)	**−0.03 (−0.05, −0.01)**	**0.004**	**−1.90 (−2.76, −1.04)**	**<0.001**	**−0.61 (−0.89, −0.32)**	**<0.001**	**−0.55 (−0.84, −0.27)**	**<0.001**

**Bold** indicates statistical significance after Benjamini-Hochberg procedure. B = coefficient and CI = confidence interval from linear regression model. Models represent the abbreviated output from the linear regression models with adjustment for age, sex, race and ethnicity, household income, parent education, attention-deficit/hyperactivity symptoms, depressive symptoms, non-social media screen time, respective baseline cognitive score, and site location.

## Discussion

In this cohort of early adolescents, we found that an increase in time spent on social media was prospectively associated with lower cognitive performance scores at the Year 2 follow-up, even after accounting for baseline cognitive performance. Analysis of the LMT, which evaluates visuospatial attention, revealed a significant association between marked increases in social media time and diminished cognitive performance scores. Similarly, for the RAVLT, which evaluates auditory verbal learning and memory recall, significant associations appeared for both small and large increases in time spent on social media and lower cognitive scores for the initial learning trials, retroactive interference, and long delay tests. Adolescents with a large increase in social media time recalled an average of approximately two fewer words in the RAVLT Initial Trials and approximately 0.6 fewer words in both the RAVLT Retroactive Interference and RAVLT Long Delay Trials, in contrast to the comparison group, who partook in no or very low social media use. These findings suggest an association between greater social media use and lower auditory verbal learning and memory recall cognitive function scores, though the effect sizes were small. Our observed associations may reflect the substantial cognitive and neurobiological changes found in early adolescence, a particularly vulnerable period for development.^40^

These findings are clinically relevant because verbal memory has been shown to accurately measure cognitive decline during the initial stages of neurocognitive impairment.^41^ In addition, deficits in visuospatial attention have been shown to have real-world implications, including associations with poorer academic performance in mathematics and reading. These deficits also heighten crash risk among novice teen drivers, as lapses in visuospatial attention may impair hazard detection and spatial judgement.^42^^,^^43^

Several potential mechanisms may explain these associations. Increased time spent on social media may displace activities that more directly support cognitive development, such as reading, studying, or engaging in in-person conversations that strengthen verbal memory and processing skills.^44^ Social media platforms may also provide a predominantly visual and fast-paced digital environment that offers fewer opportunities for sustained verbal engagement or memory consolidation.^45^ Additionally, the fragmented and often distracting nature of digital multitasking may interfere with attention and encoding processes essential for learning and recall.^46^ Future research should explore these potential pathways to better understand how digital media engagement intersects with cognitive development during adolescence.

This study adds to a growing body of evidence linking increased screen time, particularly social media use, with diminished cognitive development and capabilities in children and adolescents.^10^^,^^47^ A previous systematic review of children and adolescents aged 0–18 years found weak evidence that screen time has a negative effect on cognitive development, largely due to the lack of prior literature.^10^ Our findings contribute new insight by concentrating specifically on social media time rather than general screen time. Other research has also suggested that digital media use may be related to changes in structural brain development, such as altered cerebellar volume, though further longitudinal studies are needed to fully understand these relationships.^6^

Our findings offer novel longitudinal evidence that increasing social media use across early adolescence is prospectively associated with diminished cognitive performance. While prior studies on screen time relying on cross-sectional snapshots or limited longitudinal follow-up have yielded inconsistent findings and weak associations, our prospective analysis focusing specifically on social media time trajectories reveals stronger, more robust findings regarding these relationships. Additionally, the ABCD Study's large, diverse sample enhances generalizability, though excluded participants in the current study were more likely to be from racial/ethnic minorities or lower-income, less-educated families, which may limit the applicability of findings to these groups. Lastly, the ability to control for the same cognitive outcomes measured at baseline allows us to more precisely isolate the longitudinal effects of baseline social media and changes over time on cognitive functioning two years later.

While this study offers valuable insights, several limitations should be noted. Social media time was self-reported, introducing the potential for biases that may influence the results. Though we adjusted for potential confounders such as depressive symptoms and attention-deficit/hyperactivity symptoms, unknown or unmeasured confounding variables, such as sleep quality, nutrition, and physical activity, may still be present and potentially contribute to the observed associations. Additionally, while a large number of early adolescents report social media use and have at least one account,^48^ the average engagement time at baseline is relatively low, potentially impacting the results. Furthermore, the baseline and follow-up data were collected prior to or at the start of the COVID-19 pandemic, which doubled screen time use in the target population.^49^ The magnitude of the observed associations was small, which may reflect the relatively short follow-up from baseline to Year 2; longer-term studies may reveal stronger effects. In addition, although there is variability within the trajectory group sizes, each trajectory meets the 5% minimum membership requirement, making them statistically valid for analysis.^50^ Lastly, small subgroup sample sizes in the current study limited our ability to conduct sex-specific or race/ethnicity-specific regression analyses. Future research should seek to collect larger samples of high and increasing social media users to evaluate whether associations between social media trajectories and cognitive outcomes differ by sex or race/ethnicity.

### Conclusion

Adolescence is a period of rapid cognitive development, particularly in memory, reasoning, and decision-making.^51^ Understanding salient risk factors, such as social media use, that may hinder this development is essential for guiding clinical and policy recommendations. Active parental mediation has been shown to combat excessive social media use and reduce time online, and paediatricians can support families in implementing a ‘Family Media Use Plan,’ which can help to mitigate risks associated with media use and lessen time spent on social media.^7^^,^^52^ Our prospective findings linking greater social media use with lower cognitive scores may also shed light on the broader relationship between social media and adolescent brain development. These findings also carry relevance for clinical practice, where cognitive functioning is a key target for early intervention and developmental screening, and for health policy efforts aimed at guiding healthier social media environments for youth. Future research should explore the association between problematic social media use, rather than general use, and cognitive functioning in adolescents.

## Contributors

J.M.N. conceptualized and designed the study, curated the data, drafted the initial manuscript, critically reviewed and revised the manuscript, and had final responsibility for decisions.

J.H.W. and K.E.K. had access to the raw data, verified and analysed the data, drafted the initial manuscript, and critically reviewed and revised the manuscript.

S.N., E.J.L., and R.A.R. drafted the initial manuscript, and critically reviewed and revised the manuscript.

A.M.R., L.S., K.T.G., T.P., J.H., and A.T. critically reviewed and revised the manuscript.

All authors had access to all the data reported in the study. All authors approved the final manuscript as submitted and agree to be accountable for all aspects of the work.

## Data sharing statement

Data used in the preparation of this article were obtained from the ABCD Study (https://abcdstudy.org), held in the NIH Brain Development Cohorts (NBDC) Portal.

## Declaration of interests

The authors have no conflicts of interest to declare.
