# Supplementary material for: Associations of adolescent social media use trajectories with spatial and verbal memory: a prospective cohort study
Source: Lancet Reg Health Am. 2026 Mar 20;57:101454. doi: 10.1016/j.lana.2026.101454 (PMC13018955; doi:10.1016/j.lana.2026.101454)
Supplement: Supplementary Figure and Tables [file mmc1.pdf]

Supplementary Appendix

Nagata JM, Wong JH, Kim KE, et al. Adolescent Social Media Trajectories and Cognition: Spatial and Verbal Memory

This appendix has been provided by the authors to give readers additional information about the work

Supplementary Appendix

Table of Contents:

Figure S1. Exclusion criteria for participants in the Adolescent Brain Cognitive Development (ABCD) Study (N=7,528) Page 3

Table S1. Comparison of participants included vs excluded in the Adolescent Brain Cognitive Development (ABCD) Study at baseline (N=7,528) Page 4

Table S2. Group-based trajectory model selection Page 5

Table S3. Comparison of included participants by trajectory group in the Adolescent Brain Cognitive Development (ABCD) Study at baseline (N=7,528) Page 6

Table S4. Exclusion criteria for participants in the Adolescent Brain Cognitive Development (ABCD) Study (N=7,528) Page 7

Figure S1. Exclusion criteria for participants in the Adolescent Brain Cognitive Development (ABCD) Study (N=7,528)

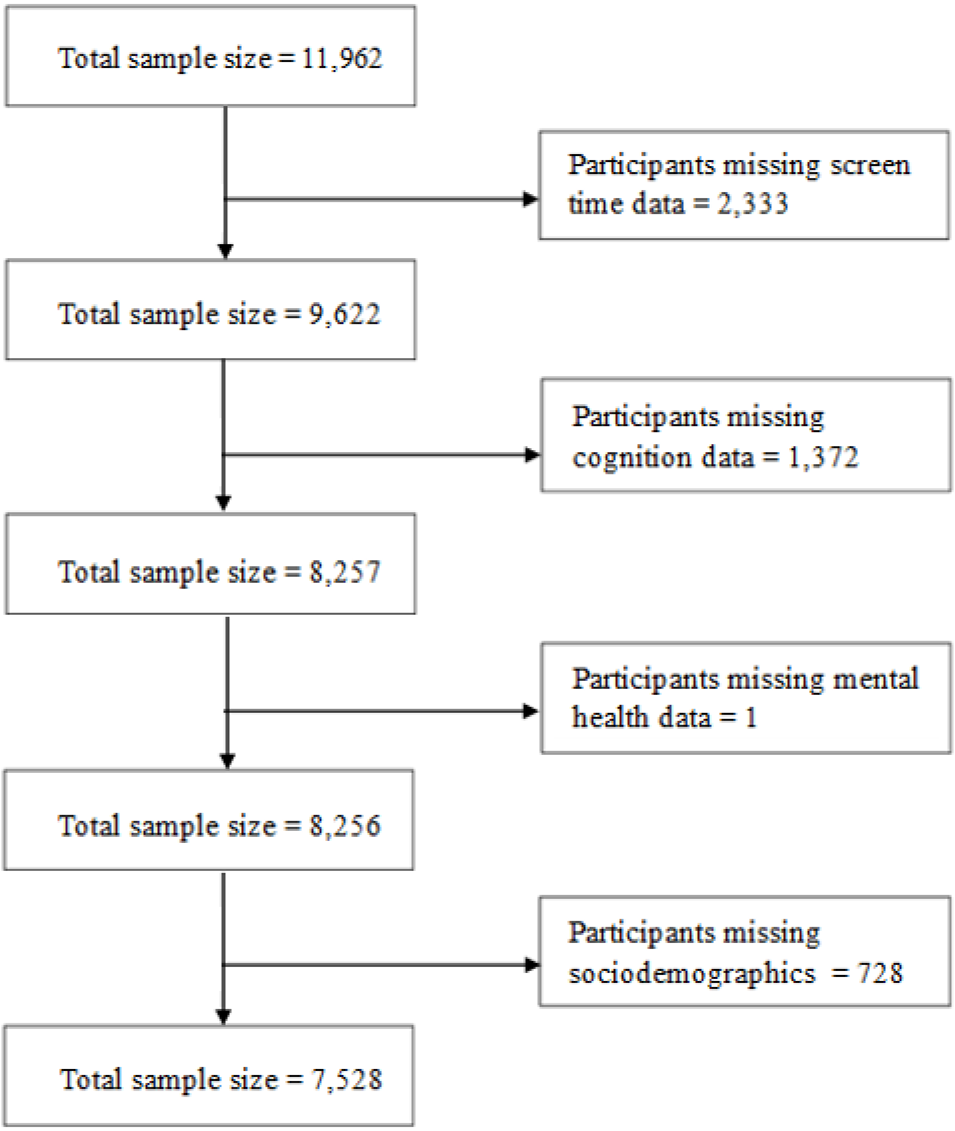

Table S1. Comparison of participants included vs excluded in the Adolescent Brain Cognitive Development (ABCD) Study at baseline (N=7,528)

Sociodemographic characteristics	Included (n=7,528)	Excluded (n=4,434)	P
Age	10.0 (0.6)	9.9 (0.6)	0.42
Sex			
Female	48.9%	48.8%	0.94
Male	51.1%	51.2%	
Race and ethnicity			
Asian	5.3%	5.8%	<0.001
Black	14.2%	22.5%	
Latino/Hispanic	18.1%	23.4%	
Native American	3.1%	3.2%	
Other	1.1%	2.0%	
White	58.2%	43.1%	
Household income			
$24,999 or less	14.9%	26.6%	<0.001
$25,000 to $49,999	19.3%	22.7%	
$50,000 to $74,999	18.3%	15.7%	
$75,000 to $99,999	14.5%	11.1%	
$100,000 to $199,999	24.9%	17.9%	
$200,000 or greater	8.1%	6.0%	
Parents' highest education			
High school education or less	14.7%	29.3%	<0.001
College education or more	85.3%	70.7%	

Table S2. Group-based trajectory model selection

Model	Trajectory (n)	APPA[1]				OCC[2]				Relative Entropy[3]	BIC[4]	Participants (%)[5]			
		Traj. 1	Traj. 2	Traj. 3	Traj. 4	Traj. 1	Traj. 2	Traj. 3	Traj. 4			Traj. 1	Traj. 2	Traj. 3	Traj. 4
Linear	2	0.96	0.86			3.08	52.60			0.82	-18197.55	87.8	12.2		
	3	0.78	0.83	0.87		2.94	7.51	110.24		0.59	-17918.20	48.2	45.2	6.6	
	4	0.74	0.81	0.67	0.87	7078.55	6.76	1.62	108.62	0.43	-17927.99	7.0	43.0	43.4	6.6
Linear with sex as the covariate	2	0.95	0.86			2.85	44.63			0.79	-18097.49	85.8	14.2		
	3	0.65	0.86	0.88		3.61	4.25	101.37		0.55	-17883.70	28.6	63.3	8.1	
	4	-[6]													
Ordered with sex as the covariate	3	0.87	0.81	0.82		113.11	3.12	7.50		0.60	-17750.74	6.4	54.7	38.8	

[1]APPA: average posterior probability of trajectory assignment, showing the average probability of participants classified into one trajectory and should be greater than 70% for all trajectories.

[2]OCC: odds of the correct classification, showing the ratio that compares the correct classification to the incorrect classification, and should be greater than 5.0.

[3]Relative entropy, with higher values indicating greater class separation.

[4]Bayesian Information Criterion (BIC), with lower values indicating better model fit.

[5]Participants (%): ≥ 5% of the sample per trajectory.

[6]Model cannot converge.

Table S3. Comparison of included participants by trajectory group in the Adolescent Brain Cognitive Development (ABCD) Study at baseline (N=7,528)

Sociodemographic characteristics	No/very low social media use (n=4,289)	Low increasing social media use (n=2,822)	High increasing social media use (n=417)	P
Age	9.9 (0.6)	10.0 (0.6)	9.8 (0.6)	0.077
Sex				
Female	38.9%	58.5%	75.2%	<0.001
Male	61.1%	41.5%	24.8%	
Race and ethnicity				
Asian	7.1%	3.4%	2.0%	<0.001
Black	9.1%	18.6%	30.3%	
Latino/Hispanic	15.3%	21.1%	23.5%	
Native American	2.8%	3.1%	6.1%	
Other	0.9%	1.3%	2.4%	
White	64.8%	52.5%	35.7%	
Household income				
$24,999 or less	10.8%	18.9%	26.3%	<0.001
$25,000 to $49,999	16.6%	21.6%	28.2%	
$50,000 to $74,999	17.7%	18.6%	22.2%	
$75,000 to $99,999	16.1%	13.3%	8.7%	
$100,000 to $199,999	29.6%	20.5%	11.9%	
$200,000 or greater	9.3%	7.1%	2.8%	
Parents' highest education				
High school education or less	10.6%	18.5%	26.4%	<0.001
College education or more	89.4%	81.5%	73.6%	

Table S4. Cognitive performance outcomes of included participants in the Adolescent Brain Cognitive Development (ABCD) Study, stratified by sex (N=7,528)

	Females (n=3,617)	Males (n=3,911)
Cognitive performance measures	Mean (SD)	Mean (SD)
Baseline measures		
Little Man Task: Accuracy	0.6 (0.2)	0.6 (0.2)
Rey Auditory Verbal Learning Test: Initial Learning Trials	45.7 (9.5)	43.9 (9.7)
Rey Auditory Verbal Learning Test: Retroactive Interference	9.9 (3.0)	9.7 (3.0)
Rey Auditory Verbal Learning Test: Long Delay Recall	9.6 (3.1)	9.1 (3.1)
Year 2 measures		
Little Man Task: Accuracy	0.7 (0.2)	0.7 (0.2)
Rey Auditory Verbal Learning Test: Initial Learning Trials	44.7 (9.1)	43.7 (9.1)
Rey Auditory Verbal Learning Test: Retroactive Interference	9.7 (2.7)	9.7 (2.8)
Rey Auditory Verbal Learning Test: Long Delay Recall	9.2 (2.9)	9.0 (3.0)

Sampling weights based on the American Community Survey were used to represent population estimates represented by the US Census.
